# Bioactivities of berberine metabolites after transformation through CYP450 isoenzymes

**DOI:** 10.1186/1479-5876-9-62

**Published:** 2011-05-15

**Authors:** Yi Li, Gang Ren, Yan-Xiang Wang, Wei-Jia Kong, Peng Yang, Yue-Ming Wang, Ying-Hong Li, Hong Yi, Zhuo-Rong Li, Dan-Qing Song, Jian-Dong Jiang

**Affiliations:** 1 Institute of Medicinal Biotechnology, Chinese Academy of Medical Sciences and Peking Union Medical College, Beijing 100050, China; 2 State Key Laboratory of Bioactive Substances and Functions of Natural Medicines, Institute of Materia Medica, Chinese Academy of Medical Sciences and Peking Union Medical College, Beijing 100050, China

## Abstract

**Background:**

Berberine (BBR) is a drug with multiple effects on cellular energy metabolism. The present study explored answers to the question of which CYP450 (Cytochrome P450) isoenzymes execute the phase-I transformation for BBR, and what are the bioactivities of its metabolites on energy pathways.

**Methods:**

BBR metabolites were detected using LC-MS/MS. Computer-assistant docking technology as well as bioassays with recombinant CYP450s were employed to identify CYP450 isoenzymes responsible for BBR phase-I transformation. Bioactivities of BBR metabolites in liver cells were examined with real time RT-PCR and kinase phosphorylation assay.

**Results:**

In rat experiments, 4 major metabolites of BBR, berberrubine (M1), thalifendine (M2), demethyleneberberine (M3) and jatrorrhizine (M4) were identified in rat's livers using LC-MS/MS (liquid chromatography-tandem mass spectrometry). In the cell-free transformation reactions, M2 and M3 were detectable after incubating BBR with rCYP450s or human liver microsomes; however, M1 and M4 were below detective level. CYP2D6 and CYP1A2 played a major role in transforming BBR into M2; CYP2D6, CYP1A2 and CYP3A4 were for M3 production. The hepatocyte culture showed that BBR was active in enhancing the expression of insulin receptor (InsR) and low-density-lipoprotein receptor (LDLR) mRNA, as well as in activating AMP-activated protein kinase (AMPK). BBR's metabolites, M1-M4, remained to be active in up-regulating InsR expression with a potency reduced by 50-70%; LDLR mRNA was increased only by M1 or M2 (but not M3 and M4) with an activity level 35% or 26% of that of BBR, respectively. Similarly, AMPK-α phosphorylation was enhanced by M1 and M2 only, with a degree less than that of BBR.

**Conclusions:**

Four major BBR metabolites (M1-M4) were identified after phase-I transformation in rat liver. Cell-free reactions showed that CYP2D6, CYP1A2 and CYP3A4 seemed to be the dominant CYP450 isoenzymes transforming BBR into its metabolites M2 and M3. BBR's metabolites remained to be active on BBR's targets (InsR, LDLR, and AMPK) but with reduced potency.

## Background

**Figure 1:**
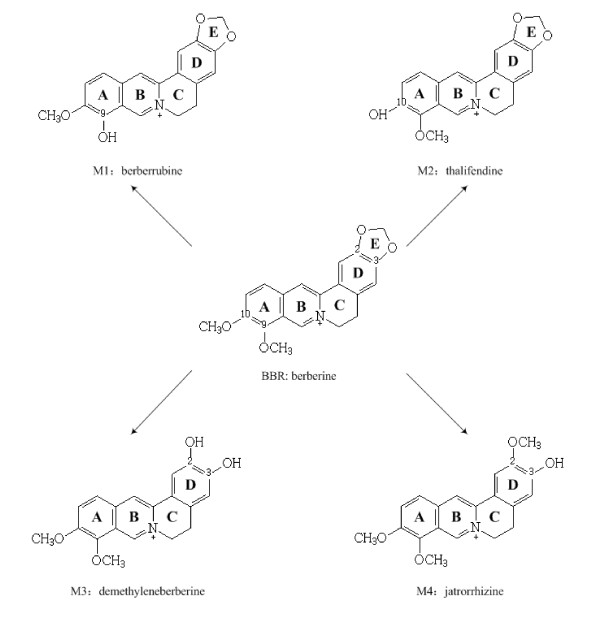
**Chemical structure of berberine and its metabolites, berberrubine (M1), thalifendine (M2), demethyleneberberine (M3) and jatrorrhizine (M4)**.

Berberine (BBR, Figure 1) is a natural compound isolated from *Coptis chinensis *and is for decades an over-the-count medicine in China for diarrhea [1]. Recently, accumulated research has identified BBR to be an effective drug in treating hyperlipidemia as well as hyperglycemia [2-4]. Clinical studies showed that oral administration of BBR caused significant reduction of blood cholesterol, triglyceride as well as glucose in patients with hyperlipidemia and type 2 diabetes [2,3,5-7], with no side-effects on liver, kidney and muscle [2,5]. Mechanism studies have identified several important modes of action involved in the activities of BBR. The cholesterol-lowering effect was associated with extracellular-signal-regulated kinase (ERK) mediated LDLR mRNA up-regulation [2,8]; the glucose-lowering effect mainly resulted from the protein kinase C (PKC) mediated InsR expression and the activation of AMPK [3,4,9,10]. The observed reduction of triglyceride by BBR might reflect its synergistic effect on both sugar and lipid metabolism [2-4].

At least 4 major metabolites of BBR have been identified in human blood after Phase I metabolism [11]; however, human CYP450 isoenzymes that are responsible for BBR phase-I transformation remain to be identified. Furthermore, bioactivities of the metabolites on the pathways mentioned above are also unknown. As most of our previous experiments for BBR were done in CYP450 positive human hepatocytes, HepG2 and Bel-7402 [2-4], we were curious to learn which of the CYP450 isoenzymes are involved in BBR phase-I metabolism, and what are the bioactivities of the BBR metabolites. Answers to these questions might be important for patient selection in BBR clinical treatment as well as for chemical modification on BBR.

## Methods

### Chemicals and reagents

Berberine (BBR) chloride (purity, 98%) was obtained from Sigma Chemical Co. (St. Louis, MO, USA). Berberrubine (M1), thalifendine (M2) and demethyleneberberine (M3) were synthesized by Chemical Department of our institute. All of the study compounds were in purity over 98.5%. Jatrorrhizine (purity, 98%) (M4) was purchased from the National Institute for the Control of Pharmaceutical and Biological Products (Beijing, China). The CYP450 inhibitors α-naphthoflavone, quinidine and ketoconazole were from J&K Chemical Ltd (Beijing, China). HPLC-grade acetonitrile and methanol were obtained from Fisher Scientific (New Jersey, USA). Pooled mixture of liver microsomes (Gentest Lot No. 70196, USA) and a NADPH-regenerating system were purchased from BD Gentest (Woburn, MA, USA). Microsomes from cDNA-transfected baculovirus-insect cells (expressing CYP1A2, CYP2A6, CYP2B6, CYP2C9, CYP2C19, CYP2D6, CYP2E1, CYP3A4 and CYP3A5) were also from the BD Gentest.

### Animal experiments

Male Wistar rats (200-240 g, 8-week-old) purchased from Vital River Laboratories (Beijing, China) fasted overnight and were free access to water before drug administration. Rats were treated with with BBR orally (200 mg/kg weight, n = 8) or distilled water as negative controls (n = 3), and rat livers were removed 3 hrs later. 2 g of the liver sample was washed with 5 mL normal saline, cut into small pieces and extracted with 3-fold volume acetonitrile. After vortexed for 3 min, the extract was centrifuged at 16000 g for 15 min at 4°C and the supernatant was diluted with 0.2% acetic acid. Ten microlitre dilution aliquot was injected into the LC-MS/MS system for analysis.

### Structure-based BBR-CYP450 docking analysis

The crystal structures of the six CYP450 isoforms were retrieved from the Protein Data Bank (PDB ID code 2HI4 for CYP1A2, solved at 1.95-Å resolution [12]; 1Z11 for CYP2A6, 2.05-Å [13]; 1OG5 for CYP2C9, 2.55-Å [14]; 2F9Q for CYP2D6, 3.00-Å [15]; 3E4E for CYP2E1, 2.60-Å [16]; 1TQN for CYP3A4, 2.05-Å [17,18]). In this study, the docking was based on steric considerations but orienting the substrate recognition sites (SRSs) toward heme and ferryl oxygen of CYP450 protein [19,20]. A docking program FlexX (SYBYL 7.3, Tripos Inc) that uses an incremental construction algorithm was applied to optimize the interaction between the ligands and the substrate binding sites [21]. As for each of the CYP450 proteins, all crystal water molecules were removed from the original structure, hydrogen was added using Biopolymer module in SYBYL.

### BBR metabolism in human liver microsomes (HLMs)

Incubation conditions were optimized to ensure the linearity with respect to the microsomal protein concentration and incubation time [22-24]. A typical incubation contained 0.1 mg of human liver microsomes, 100 mM phosphate buffer (pH 7.4), BBR (dissolved in methanol, final volume of methanol < 0.1% [25]) and NADPH-regenerating system (at final concentration of 3.3 mM glucose-6-phosphate, 1.3 mM NADP^+^, 0.4 unit/ml glucose-6-phosphate dehydrogenase and 3.3 mM MgCl_2_) in a final volume of 200 μl. After pre-incubation of HLMs with BBR for 5 min at 37°C, the reaction was initiated by addition of NADPH-regenerating system. After incubation of the mixture in a 37°C water bath with opening to the air, the reaction lasted for 30 min and was terminated by addition of 800 μl cold acetonitrile. Internal standard (40 μg/ml ampicillin, dissolved in acetonitrile) was added into the mixture, and then centrifuged at 16000 g for 15 min at 4°C. The supernatant was aspirated, followed by dilution in 0.2% acetic acid. 10 μl of the aliquot was injected into the LC-MS/MS system for analysis. Control samples were incubated without NADPH. The incubation was performed in duplicates.

### BBR metabolism in recombinant human CYP450 isoenzymes

The experiment was performed with the condition described in the method using HLMs for transformation. 50 pmol/mL of each of the CYP450 isoenzymes incubated with 20 μmol/L BBR. Control microsome prepared from insect cells, which were infected with wild-type baculovirus, severed as a negative control. The incubation was performed in duplicates.

### Chemical inhibition assays

α-Naphthoflavone [26] (typical inhibitor of CYP1A2; final concentration used, 5 μM), quinidine [27,28] (typical inhibitor of CYP2D6; final concentration, 5 μM) sulfaphenazole [23] (typical inhibitor of CYP2C9; final concentration, 5 μM), troglitazone [23] (typical inhibitor of CYP2C19; final concentration, 5 μM) and ketoconazole [29] (typical inhibitor of CYP3A; final concentration, 5 μM) were added, respectively, to the incubation reaction containing 0.5 mg/ml HLMs, 100 mM phosphate buffer (pH 7.4) and NADPH-regenerating system at a final volume of 200 μl. Before addition of BBR (15 μM), the samples were pre-incubated for 10 min at 37°C and transferred onto ice for 45 min. The mixture was incubated at 37°C for 30 min and then terminated with cold acetonitrile [30]. Sample analysis was described above. The incubations were performed in duplicates.

### Kinetic experiments

To measure the enzyme kinetic parameters both in HLMs and rCYP450s, we adjusted the standard incubation mixture containing BBR at a final concentration between 0.15~75 μM. Incubation conditions were as described above. Samples were analyzed by LC-MS/MS. The kinetic parameters Vmax and Km were calculated using EK1 v10.0 program (SPSS Inc., Chicago, IL, USA), and these values were used to calculate the intrinsic clearance (Clint, Vmax/Km). The results were expressed as mean ± sd from three independent experiemnts. The percentage of total normalized rate (%TNR) was calculated to estimate the contribution of each CYP450 in BBR transformation [22].

### LC-MS/MS analysis

Sample analysis was performed using an LC-MS/MS system (Thermo-Finnigan, San Jose, CA, USA), which consisted of a Surveyor LC pump with an on-line degasser, a Surveyor autosampler and a TSQ Quantum triple-quadrupole mass spectrometer equipped with an ESI probe. A Waters XTerra^® ^MS C_18 _(2.1 ×50 mm, 3.5 μm) column (Milford, MA, USA) was used for separation at 25°C. The mobile phase consisted of 0.2% acetic acid in water (A) and 100% acetonitrile (B). The flow-rate was 0.2 ml/min. In gradient elution, the proportion of acetonitrile (B) was linearly increased from 10% to 100% in 18 min, held at 100% for 2 min and returned to 10% in 0.1 min. The column was allowed to equilibrate for 10 min after each run. Only the data from 2 min to 15 min was acquired by MS. The MS was operated in positive ESI mode. Nitrogen was used as both the sheath and auxiliary gas at a pressure of 35 and 10 arbitrary units, respectively. The spray voltage was set at 4.0 kV and the capillary temperature was at 270°C. Full scan and product ion mass spectra results of BBR as well as M1-M4 were obtained (data are not shown). The most abundant product ion of each chemical was chosen for selected reaction monitoring (SRM). The SRM transitions and collision energies are shown in Table 1. Data were analyzed by Xcalibur 1.2 software.

**Table 1:** SRM transitions and collision energies used in LC-MS/MS for the detection of BBR, M1, M2, M3 and M4

Chemical	Molecular mass (MW)	SRM transition (*m/z*)	Collision energy (eV)
BBR	335	336.0→319.8	28
M1	321	322.0→306.8	31
M2	321	322.0→306.8	31
M3	323	324.0→307.8	32
M4	338	338.0→321.9	30

### Cell culture and drug treatment

The Human hepatoma cell line HepG2 was obtained from the American Tissue Culture Collection (ATCC, USA). HepG2 cells were grown in Eagle's Minimum Essential Medium (GIBCO) supplemented with 10% fetal bovine serum (GIBCO), 1% non-essential amino acids (GIBCO) and 1% antibiotics (100 units/ml of penicillin and 100 μg/ml of streptomycin), and incubated at 37°C in a humidified atmosphere with 5% CO_2_.

For real time RT-PCR and double immune-staining analysis, HepG2 cells were starved in Eagle's MEM supplemented with 0.5% FBS overnight and treated with BBR or its metabolites (M1-M4) for 8 hrs, respectively. For immunoblot assay, cells were starved in serum-free medium overnight and treated with BBR or its metabolites (M1-M4) for 24 hrs. The study compounds were diluted in culture medium prior to use.

### RNA isolation and real-time RT-PCR

Total cellular RNAs were isolated by the Ultraspec RNA lysis solution (Biotecxs Laboratory, Houston, TX) by following the vender's instruction. Total RNAs were reversely transcribed into cDNAs using the Reverse Transcription System (Promega, Madison, WI). Quantitative real time PCR was performed with these cDNAs as described previously [2], with β-Actin as an internal control. Normalized InsR or LDLR mRNA expression levels were plotted as fold of the untreated control. The primers used were described previously [2,3].

### Double immune-staining and flow cytometry analysis

HepG2 cells were treated with BBR (27 μM), simvastatin (1 μM) or rosiglitazone (10 μM) for 8 hrs, respectively. The cells were harvested, washed in PBS and incubated for 1 hr at 37°C in the presence of both monoclonal antibody to InsR (Labvision/NeoMarkers, Fremont, CA) and rabbit polyclonal antibody against LDLR (Santa Cruz Biotechnology, Santa Cruz, CA). The corresponding isotype-matched, nonspecific mouse and rabbit IgGs were used respectively as controls for nonspecific staining. After washing in PBS, cells were stained with a fluorescein isothiocyanate (FITC)-conjugated goat anti-mouse IgG (green color, Santa Cruz Biotechnology), as well as a tetramethyl rhodamine isothiocyanate (TRITC)-conjugated goat anti-rabbit IgG (red color, Santa Cruz Biotechnology). The fluorescence intensities were analyzed in a FACS (FACSort, Becton Dickinson).

### Immunoblot

Sample cells were rinsed with phosphate-buffered saline and lysed in SDS-PAGE loading buffer. Cell lysates were subjected to 8% SDS-PAGE for protein separation, and protein bands were transferred onto polyvinylidene difluoride membranes (Millipore). The amount of protein and extent of phosphorylation were estimated using the following primary rabbit antibodies, anti-phospho-AMPK-α-Thr-172 antibody, anti-AMPK-α antibody as well as anti-β-actin antibody (Cell signaling Technology, USA). The secondary antibody was peroxidase-conjugated goat anti-rabbit antibody (Cell signaling Technology, USA). After binding, the bands were revealed with enhanced chemiluminescence using the ECL commercial kit (Millipore).

### Statistics

Differences of mean results among study groups were examined by a two-tailed unpaired Student's *t*-test for equal or unequal variances depending on a preliminary F test for homogeneity of variance. Results are expressed as the means ± sd. P < 0.05 was considered significant.

## Results and Discussion

### BBR metabolites are detectable in rat's liver tissue

**Figure 2:**
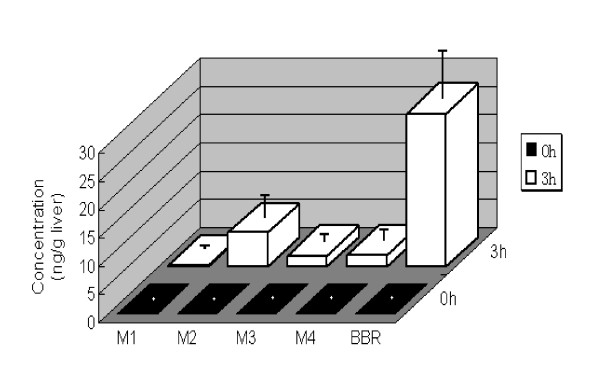
**BBR and its main phase-I metabolites in rat liver**. Male Wistar rats (200-240 g, 8-week-old) were orally treated with BBR (200 mg/kg). Rat livers (n = 8) were removed 3 hrs later, followed by homogenization, cell lysate extraction and LC MS/MS detection. Presented are mean and sd of the 8 rats. Untreated control rats (n = 8) were used for comparison.

It has been documented that BBR has four major metabolites after phase I metabolism *in vivo*, berberrubine (M1), thalifendine (M2), demethyleneberberine (M3) and jatrorrhizine (M4) [31]. The chemical structures of the four compounds are shown in Figure 1. These metabolites were detectable in the blood and urine in human and rodents [11,31]. The biotransformation of BBR in rats was reported to be similar to that in human [31]. As BBR's activity was examined in hepatocytes in our previous experiments [2-4], we detected the BBR metabolites in rat liver to address the question of whether hepatocytes contain these metabolized products. In the present study, the 4 metabolites M1, M2, M3 and M4 were all detectable in rat livers 3 hrs after BBR oral administration (Figure 2). The highest level was seen in M2 and the lowest in M1. The result was consistent with the previous reports of BBR metabolites in rodent blood [11,31].

### CYP450 isoenzymes that involved in BBR phase I metabolism

**Figure 3:**
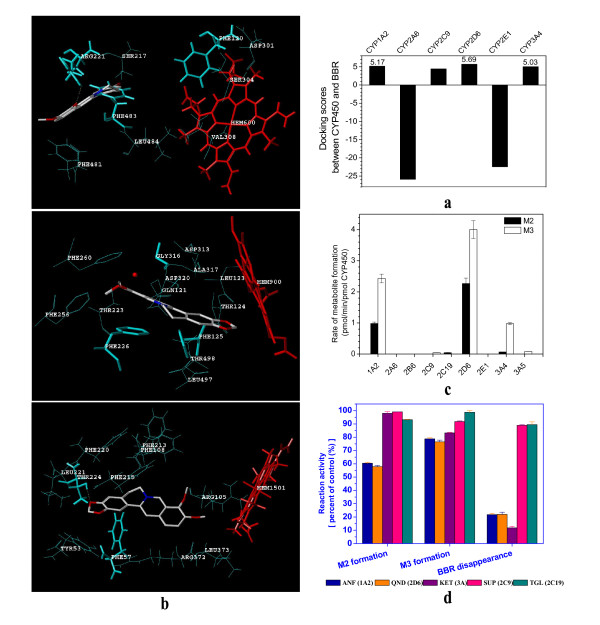
**Identification of CYP450 isoenzymes that transform BBR into its metabolites**. Interaction between CYP450 and BBR were analyzed with docking score generated from SYBYL 7.3 software analysis. CYP450 isoenzymes with docking scores over 5 were labelled with the score value (a). The 3-D structrual docking patterns between BBR and CYP2D6 (upper), or CYP1A2 (middle), or CYP3A4 (lower) were generated with SYBYL 7.3. BBR is rendered in sticks (red, oxygen atoms; white, carbon atoms; blue, nitrogen atoms). The heme prosthetic group is rendered in sticks in red. The amino acid residues constituting the active site cavity are in cyan, most of which are shown in lines. Amino acid residues rendered in sticks may help BBR binding to CYP450. Specific hydrogen bonds and a water molecule are showed as yellow broken lines and a red sphere, respectively (b). For the transformation test in rCYP450 isoenzymes reaction system, BBR (20 μM) was incubated with each of the rCYP450 isoenzymes (50 pmol/ml of rCYP450) for 0.5 hr, followed by a detection of the metabolites. The experiment was repeated twice (c). In the chemical inhibition assay, the α-naphthoflavone (ANF) was for CYP1A2 inhibition, quinidine (QND) for CYP2D6, ketoconazole (KET) for CYP3A, sulfaphenazole (SUP) for CYP2C9 and troglitazone (TGL) for CYP2C19. BBR was incubated with HLMs in the presence or absence of CYP450 specific inhibitors. The final concentration of BBR and the inhibitor in the reaction was 15 μM and 5 μM, respectively. The enzyme catalyzing activity of sample free of inhibitors (control) was defined as 100%. Presented is mean and sd of the percent of control (d).

For BBR's metabolite formation in liver, our question was which of the CYP450 isoenzymes transform BBR into its metabolites. Thus, nine major recombinant human CYP450 isoenzymes (CYP1A2, CYP2A6, CYP2B6, CYP2C9, CYP2C19, CYP2D6, CYP2E1, CYP3A4 and CYP3A5) were investigated in this study. Of the nine isoenzymes, six (CYP1A2, CYP2A6, CYP2C9, CYP2D6, CYP2E1 and CYP3A4) have their 3-D structural database available from PDB (Protein Data Bank). As the initial step, computer-assistant docking analysis was performed using SYBYL 7.3 system for the affinity between BBR and each isoenzyme. As shown in Figure 3a for the docking score, the CYP2D6, CYP1A2 and CYP3A4 exhibited considerably good docking performance with respect to the positive docking cut-off value set at 5. The molecular interactions between BBR and the three CYP450s are shown in Figure 3b. BBR anchored in the binding site of CYP2D6 through hydrogen bond interaction with the side chain of Arg-221; Phe-120 played a major role in controlling the orientation of BBR to the heme; the Phe-483 made a cross lock to the C- and D-ring of BBR (Figure 3b, **upper**). The substrate binding cavity of CYP1A2 was uniformly narrow throughout its extent and was lined by residues Gly-316-Ala-317 and the Asp-320 peptide bond, which constituted a relatively planar substrate binding platform. Phe-226 produced another parallel substrate binding surface and a π-π stacking with BBR. In addition, both orthogonal and parallel aromatic interactions between BBR and residues Phe-125 and Phe-226 contributed to a tight binding affinity. Also, a water molecule of CYP1A2 hydrogen-bonding to BBR and Gly-316 strengthens this binding (Figure 3b, **middle**). In the BBR-CYP3A4 docking pattern, hydrogen bonding and orthogonal aromatic interactions were observed between BBR and the residues Thr-224 and Phe-57, respectively (Figure 3b, **lower**). The results suggest that the three cellular CYP450 isoenzymes might have good binding affinity to BBR.

Then, the docking experiment was corroborated with biochemical assay. Each of the above-mentioned CYP450 isoenzymes was in turn incubated with BBR for 30 min, followed by detection of BBR metabolites with LC-MS/MS. The results are shown in Figure 3c. Treatment of BBR with CYP2D6 and CYP1A2 generated a considerable level of M2 and M3, suggesting that they were the dominant contributors to transform BBR into M2 and M3. CYP3A4 was also important for the production of M3 but only with a minor role in M2 production. In addition, CYP2C19, CYP2C9 and CYP3A5 showed some very weak effect in producing M2 and M3, respectively. CYP2A6, CYP2B6 and CYP2E1 were without effect on BBR transformation. Although the experiment was repeated for over 5 times, M1 and M4 were not detectable in the reaction system.

If the results were true, inhibition of the CYP450 isoenzymes should reduce the formation of the metabolites. Therefore, chemical inhibition assays were carried out in the HLMs reaction system for the CYP2D6-, CYP1A2-, CYP3A-, CYP2C9-, or CYP2C19-mediated BBR transformation, respectively. BBR was treated with HLMs for 30 min in the presence or absence of specific CYP450 isoenzyme inhibitors. The effects of the inhibitors on BBR metabolism in HLMs reaction were shown in Figure 3d. The reaction activity of the control sample (with no inhibitor) was defined as 100% for the comparison with that of the samples treated with inhibitors. M2 formation was decreased by about 40% after ANF (for CYP1A2) or QND (for CYP2D6) treatment, but not KET treatment (for CYP3A); M3 formation decreased by around 20% after treatment with inhibitor for CYP1A2, or CYP2D6, or CYP3A; inhibitors for CYP2C9 and CYP2C19 showed only minor or no effect. Accordingly, BBR disappearance was found decreased in the reactions treated with inhibitors for CYP1A2, CYP2D6 and CYP3A, but not with those for CYP2C9 and CYP2C19. The sum of reduction in the formation of M2 and M3 were close to the reduction of berberine disappearance (Figure 3d). The results showed the role of the three CYP450 isoenzymes in the BBR phase-I metabolism, and were consistent with that from computer docking. The inhibition rate of each of the inhibitors for CYP1A2, CYP2D6 and CYP3A was in the range between 15-40%. The inhibitory efficacy of each of the inhibitors was not strong (especially for the inhibition of M3 formation), indicating a combined effect of the three major isoenzymes in BBR's transformation.

Considering the relative expression of these CYP450 isoforms in liver, enzyme kinetic study using these CYP450 isoenzymes was performed and the relative contribution of each of the CYP450 isoenzymes was calculated. The results were in Table 2 and 3. For M2 production, the contribution rate of CYP1A2, CYP2D6 and CYP3A4 was 72.07%, 25.21% and 2.72%, respectively. For M3, the contribution rate was 26.36%, 37% and 36.63% for CYP1A2, CYP2D6 and CYP3A4, respectively.

**Table 2:** CYP450s responsible for M2 formation

	CYP1A2	CYP2D6	CYP3A4
Km (μM)	100.0 ± 8.74	31.9 ± 1.23	27.8 ± 0.79
Vmax (pmol/min/pmol P450)	5.4 ± 0.094	2.7 ± 0.0052	0.024 ± 0.0021
Clint (μL/min/pmol P450)	0.054	0.085	0.00085
Nominal P450 Content in HLMs (pmol/mg)	45	10	108
Adjusted Clint (μL/min/mg)	2.43	0.85	0.0918
Percentage Adjusted Clint (%)	72.07	25.21	2.72

**Table 3:** CYP450s responsible for M3 formation

	CYP1A2	CYP2D6	CYP3A4
Km (μM)	125.8 ± 3.57	12.0 ± 0.68	27.1 ± 1.03
Vmax (pmol/min/pmol P450)	4.8 ± 0.54	2.9 ± 0.19	0.59 ± 0.012
Clint (μL/min/pmol P450)	0.038	0.24	0.022
Nominal P450 Content in HLMs (pmol/mg)	45	10	108
Adjusted Clint (μL/min/mg)	1.71	2.4	2.376
Percentage Adjusted Clint (%)	26.36	37.0	36.63

### Activity of the BBR metabolites on InsR, LDLR and AMPK

**Figure 4:**
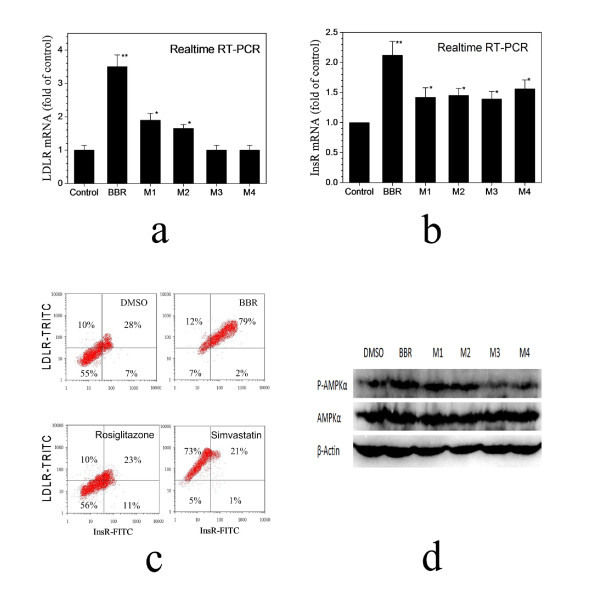
**Effect of BBR metabolites on InsR, LDLR and AMPK**. HepG2 cells were respectively treated with the compounds for 8 hrs at concentration of 20 μM for InsR test or 40 μM for LDLR, followed by RNA extraction and real time RT-PCR assay for the InsR or LDLR mRNA. The amount of LDLR (**a**) and InsR (**b**) mRNA in the treated cells was normalized to that of the untreated control. * p < 0.05, **p < 0.01, vs the untreated control. To detect the protein expression, HepG2 cells were treated with BBR (27 μM) for 8 hrs, with simvastatin (1 μM) and rosiglitazone(10 μM) as references. Berberine increased the cell-surface expression of both InsR and LDLR in the hepatocytes (**c**). For AMPK activation, HepG2 cells were treated with the study compounds (20 μM) for 24 hrs with DMSO as a control. AMPK-alpha phosphorylation in the HepG2 cells was detected with immunoblots, using a protocol described in the Methods (**d**).

Next, each of the BBR metabolite compounds was examined for their effect on the expression of InsR and LDLR mRNA, as well as on the activity of AMPK in human hepatocytes. For LDLR mRNA expression, the original BBR remained to be the strongest up-regulator (Figure 4a); among the 4 metabolites, M1 and M2 remained to be active with an activity 35% and 26% of that of BBR, respectively; M3 and M4 were without effect. Although the 4 metabolites were active in increasing InsR mRNA level in HepG2 hepatocytes by around 1.5 folds, the original form of BBR appeared to be the most active one up-regulating InsR mRNA level by over 2 folds (Figure 4b). It suggests that BBR in its original form is the most potent functional compound for the hypoglycemic as well as lipid-lowering effect in clinic. To compare BBR's effect with the known type 2 diabetes drug rosiglitazone and anti-cholesterol drug simvastatin, double immune-staining was conducted for the cell-surface protein expression of InsR and LDLR. The results demonstrated an up-regulatory effect of BBR on both LDLR and InsR on the membrane of human hepatocytes, suggesting a unique effect of BBR on the two major molecules related to energy metabolism. In contrast, simvastatin increased LDLR expression only, but not InsR; and rosiglitazone showed effect on neither LDLR nor InsR (Figure 4c). The results hint an advantage of BBR over the known drugs in modulating cellular energy metabolism.

AMPK's activity closely associates with sugar metabolism. Kim et al has reported that BBR reduced blood glucose through activation of AMPK in adipose cells [9]. Here, we tested BBR metabolites for their activity on this kinase in liver cells. As shown in Figure 4d, among the study compounds BBR was again the most active compound for AMPK activation; the treatment of HepG2 human liver cells with M1 and M2 increased the activity of AMPK but with a less potency as compared to that of BBR; the effect of M3 and M4 on this kinase appeared to be not significant. The experiment was repeated for more than 3 times and the results were reproducible. As the BBR bioactivities were kept at certain degrees in the metabolites with an intact E ring (M1 and M2), but abolished in those with E ring opened (M3 and M4), the presence of E ring on BBR (Figure 1) seems to be essential for their activity on LDLR and AMPK, consistent with our previous observation [32]. The analysis of the functional group of BBR has led us to a creation of an M1 pro-drug, which demonstrated an increased efficacy as compared with BBR [33].

BBR is a safe medicine with multiple therapeutic effects. This compound has a poor absorption rate in human gut with less than 10% of BBR entering into blood stream [34]. BBR were metabolized into at least four metabolites after phase-I metabolism *in vivo*. This study shows for the first time that CYP1A2, CYP2D6 and CYP3A4 are the major contributors for the transformation of BBR into its metabolites in human liver microsomes. The main metabolites detectable in the enzymatic reaction were M2 and M3; M1 and M4 were not found after incubation of BBR with recombinant CYP450s or the HLMs mixture. As the sensitivity of our LC-MS/MS test was in 100~500 pg range, the M1 and M4 content might be under this level in the reactions. Further optimization of the experimental condition might help us to identify these two metabolites although their concentration seemed to be extremely low in these reactions. It is worthy to note that statins are mainly metabolized under the action of CYP3A4 [35,36], relatively different from that in BBR. The information might be helpful in designing clinical therapeutic regimen combining statins with BBR [7].

Also, this study shows for the first time that although BBR itself is the most active compound to up-regulate InsR expression, all of its metabolites remain to be active at certain degree on this target. For LDLR up-regulation and AMPK activation, M1 and M2 are the two active metabolites, with reduced potency. The study suggests that the action of BBR on cellular energy metabolism pathways mainly originates from BBR in its original form.

## Conclusions

Taken together, four major BBR metabolites (M1-M4) were identified after phase-I transformation in rat liver. CYP1A2, CYP2D6 and CYP3A4 appeared to be the key enzymes to transform BBR into M2 and M3 in cell-free metabolism reactions. The metabolites of BBR remained to be active on InsR (by M1-4), LDLR (by M1 and M2), and AMPK (by M1 and M2), but with largely reduced potency. It appears that BBR in its original form is the active chemical entity that regulates multiple pathways in energy metabolism.

## List of abbreviations

BBR: berberine; M1: berberrubine; M2: thalifendine; M3: demethyleneberberine: M4: jatrorrhizine; HLMs: human liver microsomes; rCYP450: recombinant human cytochrome P450; LC-MS/MS: liquid chromatography - tandem mass spectrometry; NADPH: reduced nicotinamide adenine dinucleotide phosphate; InsR: insulin receptor; LDLR: low-density-lipoprotein-receptor; AMPK: AMP-activated protein kinase; ERK: extracellular-signal-regulated kinase; PKC: protein kinase C.

## Competing interests

The authors declare that they have no competing interests.

## Authors' contributions

LY carried out the CYP450 metabolism study, computer-aided docking study, rat experiment and participated in writing of the manuscript; RG carried out the AMPK activity assay and participated in writing of the manuscript; KWJ carried out the LDLR and InSR expression analysis; YP, WYX and LYH did the synthesis and analysis of M1, M2 and M3; YH and LZR participated in the computer-aided design assay; JJD and SDQ designed and organized the studies, analysed data and wrote the manuscript. All of the authors read and approved the final manuscript.

## References

[B1] Zhao GP Zhao GP Huanglian Chinese Materia Medica Dictionary 2006 2 2 Shanghai: Science and Technology Press 2815 2820

[B2] Kong WJ Wei J Abidi P Lin M Inaba S Li C Wang Y Wang Z Si S Pan H Wang S Wu J Wang Y Li Z Liu J Jiang JD Berberine is a Novel Cholesterol-Lowering Drug Working Through a Unique Mechanism Distinct from Stains Nat Med 2004 10 1344 1351 10.1038/nm1135 15531889

[B3] Zhang H Wei J Xue R Wu JD Zhao W Wang ZZ Wang SK Zhou ZX Song DQ Wang YM Pan HN Kong WJ Jiang JD Berberine lowers blood glucose in type 2 diabetes mellitus patients through increasing insulin receptor expression Metabolism 2010 59 285 292 10.1016/j.metabol.2009.07.029 19800084

[B4] Kong WJ Zhang H Song DQ Xue R Zhao W Wei J Wang YM Shan N Zhou ZX Yang P You XF Li ZR Si SY Zhao LX Pan HN Jiang JD Berberine reduces insulin resistance through protein kinase C-dependent up-regulation of insulin receptor expression Metabolism 2009 58 109 119 10.1016/j.metabol.2008.08.013 19059538

[B5] Zhao W Xue R Zhou ZX Kong WJ Jiang JD Reduction of blood lipid by berberine in hyperlipidemic patients with chronic hepatitis or liver cirrhosis Biomed Pharmacother 2008 62 730 731 10.1016/j.biopha.2008.01.007 18337056

[B6] Zhang Y Li X Zou D Liu W Yang J Zhu N Huo L Wang M Hong J Wu P Ren G Ning G Treatment of Type 2 Diabetes and Dyslipidemia with the Natural Plant Alkaloid Berberine J Clin Endocrinol Metab 2008 93 2559 2265 10.1210/jc.2007-2404 18397984

[B7] Kong WJ Wei J Zuo ZY Wang YM Song DQ You XF Zhao LX Pan HN Jiang JD Combination of simvastatin with berberine improves the lipid-lowering efficacy Metabolism 2008 57 1029 1037 10.1016/j.metabol.2008.01.037 18640378

[B8] Abidi P Zhou Y Jiang JD Liu J Extracellular signal-regulated kinase-dependent stabilization of hepatic low-density lipoprotein receptor mRNA by herbal medicine berberine Arterioscler Thromb Vasc Biol 2005 25 2170 2176 10.1161/01.ATV.0000181761.16341.2b 16100034

[B9] Lee YS Kim WS Kim KH Yoon MJ Cho HJ Shen Y Ye JM Lee CH Oh WK Kim CT Hohnen-Behrens C Gosby A Kraegen EW James DE Kim JB Berberine, a natural plant product, activates AMP-activated protein kinase with beneficial metabolic effects in diabetic and insulin-resistant states Diabetes 2006 55 2256 2264 10.2337/db06-0006 16873688

[B10] Yin J Gao Z Liu D Liu Z Ye J Berberine improves glucose metabolism through induction of glycolysis Am J Physiol Endocrinol Metab 2008 294 E148 E156 17971514 10.1152/ajpendo.00211.2007 PMC2464622

[B11] Zuo F Nakamura N Akao T Hattori M Pharmacokinetics of berberine and its main metabolites in conventional and pseudo germ-free rats determined by liquid chromatography/ion trap mass spectrometry Drug Metab Dispos 2006 34 2064 2072 10.1124/dmd.106.011361 16956957

[B12] Sansen S Yano JK Reynald RL Schoch GA Griffin KJ Stout CD Johnson EF Adaptations for the Oxidation of Polycyclic Aromatic Hydrocarbons Exhibited by the Structure of Human P450 1A2 J Biol Chem 2007 282 14348 1455 10.1074/jbc.M611692200 17311915

[B13] Sansen S Hsu MH Stout CD Johnson EF Structural insight into the altered substrate specificity of human cytochrome P450 2A6 mutants Arch Biochem Biophys 2007 464 197 206 10.1016/j.abb.2007.04.028 17540336 PMC2773796

[B14] Wester MR Yano JK Schoch GA Yang C Griffin KJ Stout CD Johnson EF The Structure of Human Cytochrome P450 2C9 Complexed with Flurbiprofen at 2.0-A Resolution J Biol Chem 2004 279 35630 35637 10.1074/jbc.M405427200 15181000

[B15] Rowland P Blaney FE Smyth MG Jones JJ Leydon VR Oxbrow AK Lewis CJ Tennant MG Modi S Eggleston DS Chenery RJ Bridges AM Crystal structure of human cytochrome P450 2D6 J Biol Chem 2006 281 7614 7622 10.1074/jbc.M511232200 16352597

[B16] Porubsky PR Meneely KM Scott EE Structures of human cytochrome P450 2E1: insights into the binding of inhibitors and both small molecular weight and fatty acid substrates J Biol Chem 2008 283 33698 33707 10.1074/jbc.M805999200 18818195 PMC2586265

[B17] Ekroos M Sjögren T Structural basis for ligand promiscuity in cytochrome P450 3A4 Proc Natl Acad Sci USA 2006 103 13682 13687 10.1073/pnas.0603236103 16954191 PMC1564212

[B18] Yano JK Wester MR Schoch GA Griffin KJ Stout CD Johnson EF The structure of human microsomal cytochrome P450 3A4 determined by X-ray crystallography to 2.05-A resolution J Biol Chem 2004 279 38091 38094 10.1074/jbc.C400293200 15258162

[B19] Szklarz GD Halpert JR Molecular basis of P450 inhibition and activation implications for drug development and drug therapy Drug Metab Dispos 1998 26 1179 1184 9860924

[B20] Gotoh O Cytochrome P450 Family 2 (CYP2) Proteins inferred from Comparative Analyses of Amino Acid and Coding Nucleotide Sequences J Biol Chem 1992 267 83 90 1730627

[B21] Fu DH Jiang W Zheng JT Zhao GY Li Y Yi H Li ZR Jiang JD Yang KQ Wang Y Si SY Jadomycin B, an Aurora-B kinase inhibitor discovered through virtual screening Mol Cancer Ther 2008 7 2386 2393 10.1158/1535-7163.MCT-08-0035 18723485

[B22] Rodrigues AD Integrated cytochrome P450 reaction phenotyping: attempting to bridge the gap between cDNA-expressed cytochromes P450 and native human liver microsomes Biochem Pharmacol 1999 57 465 480 9952310 10.1016/s0006-2952(98)00268-8

[B23] Lu AY Wang RW Lin JH Cytochrome P450 in vitro reaction phenotyping: a re-evaluation of approaches used for P450 isoform identification Drug Metab Dispos 2003 31 345 350 10.1124/dmd.31.4.345 12642457

[B24] Bjornsson TD Callaghan JT Einolf HJ Fischer V Gan L Grimm S Kao J King SP Miwa G Ni L Kumar G McLeod J Obach RS Roberts S Roe A Shah A Snikeris F Sullivan JT Tweedie D Vega JM Walsh J Wrighton SA The conduct of in vitro and in vivo drug-drug interaction studies a Pharmaceutical Research and Manufacturers of America (PhRMA) perspective Drug Metab Dispos 2003 21 815 832 10.1124/dmd.31.7.815 12814957

[B25] Hickman D Wang JP Wang Y Unadkat JD Evaluation of the selectivity of In vitro probes and suitability of organic solvents for the measurement of human cytochrome P450 monooxygenase activities Drug Metab Dispos 1998 26 207 215 9492382

[B26] Koley AP Buters JT Robinson RC Markowitz A Friedman FK Differential mechanisms of cytochrome P450 inhibition and activation by alpha-naphthoflavone J Biol Chem 1997 272 3149 3152 10.1074/jbc.272.6.3149 9013547

[B27] McLaughlin LA Paine MJ Kemp CA Maréchal JD Flanagan JU Ward CJ Sutcliffe MJ Roberts GC Wolf CR Why is quinidine an inhibitor of cytochrome P450 2D6? The role of key active-site residues in quinidine binding J Biol Chem 2005 280 38617 38624 10.1074/jbc.M505974200 16162505

[B28] Guengerich FP Miller GP Hanna IH Sato H Martin MV Oxidation of methoxyphenethylamines by cytochrome P450 2D6. Analysis of rate-limiting steps J Biol Chem 2002 277 33711 33719 10.1074/jbc.M205146200 12093814

[B29] Baune B Furlan V Taburet AM Farinotti R Effect of Selected Antimalarial Drugs and Inhibitors of Cytochrome P-450 3A4 on Halofantrine Metabolism by Human Liver Microsomes Drug Metab Dispos 1999 27 565 568 10220483

[B30] Murphy PJ The Development of Drug Metabolism Research as Expressed in the Publications of ASPET: Part 3, 1984-2008 Drug Metab Dispos 2008 36 1977 1982 10.1124/dmd.108.023226 18635745

[B31] Qiu F Zhu Z Kang N Piao S Qin G Yao X Isolation and identification of urinary metabolites of berberine in rats and humans Drug Metab Dispos 2008 36 2159 2165 10.1124/dmd.108.021659 18703644

[B32] Li YH Yang P Kong WJ Wang YX Hu CQ Zuo ZY Wang YM Gao H Gao LM Feng YC Du NN Liu Y Song DQ Jiang JD Berberine analogues as a novel class of the low-density-lipoprotein receptor up-regulators: synthesis, structure-activity relationships, and cholesterol-lowering efficacy J Med Chem 2009 52 492 501 10.1021/jm801157z 19090767

[B33] Li YH Li Y Yang P Kong WJ You XF Ren G Deng HB Wang YM Wang YX Jiang JD Song DQ Design, synthesis, and cholesterol-lowering efficacy for prodrugs of berberrubine Bioorg Med Chem 2010 18 17 6422 8 10.1016/j.bmc.2010.06.106 20673726

[B34] Hua W Ding L Chen Y Gong B He J Xu G Determination of berberine in human plasma by liquid chromatography-electrospray ionization-mass spectrometry J Pharm Biomed Anal 2007 44 931 937 10.1016/j.jpba.2007.03.022 17531424

[B35] Bellosta S Paoletti R Corsini A Safety of Statins: Focus on Clinical Pharmacokinetics and Drug Interactions Circulation 2004 109 III50 III7 15198967 10.1161/01.CIR.0000131519.15067.1f

[B36] Chasman DI Posada D Subrahmanyan L Cook NR Stanton VP Jr Ridker PM Pharmacogenetic study of statin therapy and cholesterol reduction Jama 2004 291 2821 2827 10.1001/jama.291.23.2821 15199031

